# Development of a Predictive Model for Metabolic Syndrome Using Noninvasive Data and its Cardiovascular Disease Risk Assessments: Multicohort Validation Study

**DOI:** 10.2196/67525

**Published:** 2025-05-02

**Authors:** Jin-Hyun Park, Inyong Jeong, Gang-Jee Ko, Seogsong Jeong, Hwamin Lee

**Affiliations:** 1 Korea University College of Medicine Seoul Republic of Korea; 2 Korea University Guro Hospital Seoul Republic of Korea

**Keywords:** metabolic syndrome prediction, noninvasive data, clinical interpretable model, body composition data, early intervention

## Abstract

**Background:**

Metabolic syndrome is a cluster of metabolic abnormalities, including obesity, hypertension, dyslipidemia, and insulin resistance, that significantly increase the risk of cardiovascular disease (CVD) and other chronic conditions. Its global prevalence is rising, particularly in aging and urban populations. Traditional screening methods rely on laboratory tests and specialized assessments, which may not be readily accessible in routine primary care and community settings. Limited resources, time constraints, and inconsistent screening practices hinder early identification and intervention. Developing a noninvasive and scalable predictive model could enhance accessibility and improve early detection.

**Objective:**

This study aimed to develop and validate a predictive model for metabolic syndrome using noninvasive body composition data. Additionally, we evaluated the model’s ability to predict long-term CVD risk, supporting its application in clinical and public health settings for early intervention and preventive strategies.

**Methods:**

We developed a machine learning–based predictive model using noninvasive data from two nationally representative cohorts: the Korea National Health and Nutrition Examination Survey (KNHANES) and the Korean Genome and Epidemiology Study. The model was trained using dual-energy x-ray absorptiometry data from KNHANES (2008-2011) and validated internally with bioelectrical impedance analysis data from KNHANES 2022. External validation was conducted using Korean Genome and Epidemiology Study follow-up datasets. Five machine learning algorithms were compared, and the best-performing model was selected based on the area under the receiver operating characteristic curve. Cox proportional hazards regression was used to assess the model’s ability to predict long-term CVD risk.

**Results:**

The model demonstrated strong predictive performance across validation cohorts. Area under the receiver operating characteristic curve values for metabolic syndrome prediction ranged from 0.8338 to 0.8447 in internal validation, 0.8066 to 0.8138 in external validation 1, and 0.8039 to 0.8123 in external validation 2. The model’s predictions were significantly associated with future cardiovascular risk, with Cox regression analysis indicating that individuals classified as having metabolic syndrome had a 1.51-fold higher risk of developing CVD (hazard ratio 1.51, 95% CI 1.32-1.73; *P*<.001). The ability to predict long-term CVD risk highlights the potential utility of this model for guiding early interventions.

**Conclusions:**

This study developed a noninvasive predictive model for metabolic syndrome with strong performance across diverse validation cohorts. By enabling early risk identification without laboratory tests, the model enhances accessibility in primary care and large-scale screenings. Its ability to predict long-term CVD risk supports proactive intervention strategies, potentially reducing the burden of cardiometabolic diseases. Further research should refine the model with additional clinical factors and broader population validation to maximize its clinical impact.

## Introduction

Metabolic syndrome is characterized by a combination of metabolic abnormalities, such as dyslipidemia, hypertension, insulin resistance, and abdominal obesity [1,2]. Together, these factors increase the risk of cardiovascular diseases (CVD) and other chronic conditions [3]. Metabolic syndrome affects a significant portion of the global population, with increasing prevalence rates, especially in aging and urbanized populations [4]. According to the National Cholesterol Education Program Adult Treatment Panel III criteria, metabolic syndrome is diagnosed when at least three of the following five risk factors are present: abdominal obesity, elevated triglyceride levels, low high-density lipoprotein (HDL) levels, hypertension, and high fasting blood glucose [5].

Prolonged exposure to these risk factors significantly increases the likelihood of developing CVD, such as myocardial infarction, ischemic stroke, and atherosclerosis [6]. Numerous studies have established a strong link between metabolic syndrome and CVD [2], indicating that the early identification and management of metabolic syndrome may play a critical role in preventing CVD [7].

In recent years, there has been growing interest in using machine learning and deep learning models to predict metabolic syndrome and related chronic diseases, such as CVD [8,9]. These models aim to facilitate early identification and intervention, thereby potentially reducing the risk of CVD in individuals with metabolic syndrome. However, many existing prediction models rely heavily on biochemical data obtained from invasive tests such as blood samples and lab-based measurements [10,11], which limits their applicability in public health centers and large-scale health management programs.

To overcome these limitations, recent efforts have shifted toward developing metabolic syndrome prediction models using noninvasive data [12] such as demographic factors and medical history, which are more accessible and feasible in both clinical and nonclinical settings. Despite these advancements, significant challenges remain, including limitations in the diversity and size of data samples, concerns about the reliability of self-reported survey data, and a lack of external validation to confirm the generalizability of these models. Additionally, many models experience the black-box problem, often limiting their usefulness as they only provide binary predictions, making their integration into health care workflows difficult [13-15].

This study develops a predictive model for metabolic syndrome using noninvasive body composition data, enabling early detection without reliance on laboratory tests. By enhancing accessibility in primary care, public health centers, and even nonclinical settings, this approach facilitates large-scale screening and timely intervention. Furthermore, we assess the model’s ability to predict long-term CVD risk, reinforcing its potential to identify high-risk individuals early and optimize preventive strategies. This study addresses a critical gap in current screening methods by providing a practical, scalable tool for metabolic syndrome and CVD risk assessment in real-world health care settings.

## Methods

### Study Population and Data Sources

The Korea National Health and Nutrition Examination Survey (KNHANES) is a continuous surveillance system designed to assess the health and nutritional status of the Korean population, monitor trends in health risk factors and the prevalence of major chronic diseases, and provide data for the development and evaluation of health policies and programs in Korea. The surveillance was conducted by the Korea Center for Disease Control and Prevention. The KNHANES is an annual nationwide cross-sectional survey that targets a nationally representative sample of noninstitutionalized civilians in Korea. The survey comprised three components: health interviews, health examinations, and nutrition surveys. One week after the health interview and examination, a nutritionist visited the participants’ homes to conduct the nutrition survey. The survey collected detailed information on socioeconomic status, health behaviors, quality of life, health care utilization, anthropometric measurements, biochemical profiles using fasting serum and urine samples, dental health, vision and hearing assessments, bone density measurements, x-ray results, and dietary intake and eating behaviors [16].

The Korean Genome and Epidemiology Study (KoGES) began in 2001 and aimed to assess the effects of dietary, environmental, and lifestyle determinants on the development of chronic diseases, such as diabetes, hypertension, and metabolic syndrome, in the general Korean population. Between 2001 and 2002, 10,030 Korean adults aged 40-69 years from the urban city of Ansan and the rural city of Anseong were recruited at baseline. All participants completed interviewer-administered questionnaires covering demographic information and lifestyle factors, including dietary habits, health status, and medical history, and underwent anthropometric measurements and biochemical examinations every 2 years [17].

Dual-energy x-ray absorptiometry (DEXA) and bioelectrical impedance analysis (BIA) are commonly used for body composition analyses. DEXA is widely recognized as the gold standard method for body composition analysis owing to its accuracy, whereas BIA offers advantages in terms of ease of use and accessibility [18]. Although some differences in the measurement outcomes between DEXA and BIA have been reported [19,20], we aimed to develop a model that predicts metabolic syndrome quickly, conveniently, and accurately. Therefore, in this study, DEXA data were used for internal model development to leverage its accuracy as the gold standard method, whereas BIA data were used for external validation to assess the generalizability of the model across different measurement techniques. This approach allowed us to evaluate the practical applicability of the model in more accessible clinical settings where DEXA may not be available, thus ensuring the relevance and utility of the model in a wider range of health care environments.

Data from the 2008 to 2011 KNHANES, measured using DEXA, were used for model development. Data from the 2022 KNHANES were used for internal validation. KoGES data measured using BIA (InBody 720 and 970) were used for external validation, with the first and second follow-up data used for the first and second external validations, respectively.

### Ethical Considerations

The KNHANES data were approved by the Institutional Review Board of the Korea Center for Disease Control and Prevention for the years 2008-2011 and 2022. For the KoGES data, participation in the study was voluntary, and informed consent was obtained from all participants for each baseline and follow-up survey. Ethics approval for the study protocol and data analysis was granted by the Institutional Review Board of Korea University Guro Hospital (2023GR0293). Additionally, the data were anonymized before use, and all processes—including data collection, processing, and model development—adhered to the guidelines established for machine learning model development in the biomedical field [21].

### Cohort Formation and Exclusion Criteria

**Figure 1:**
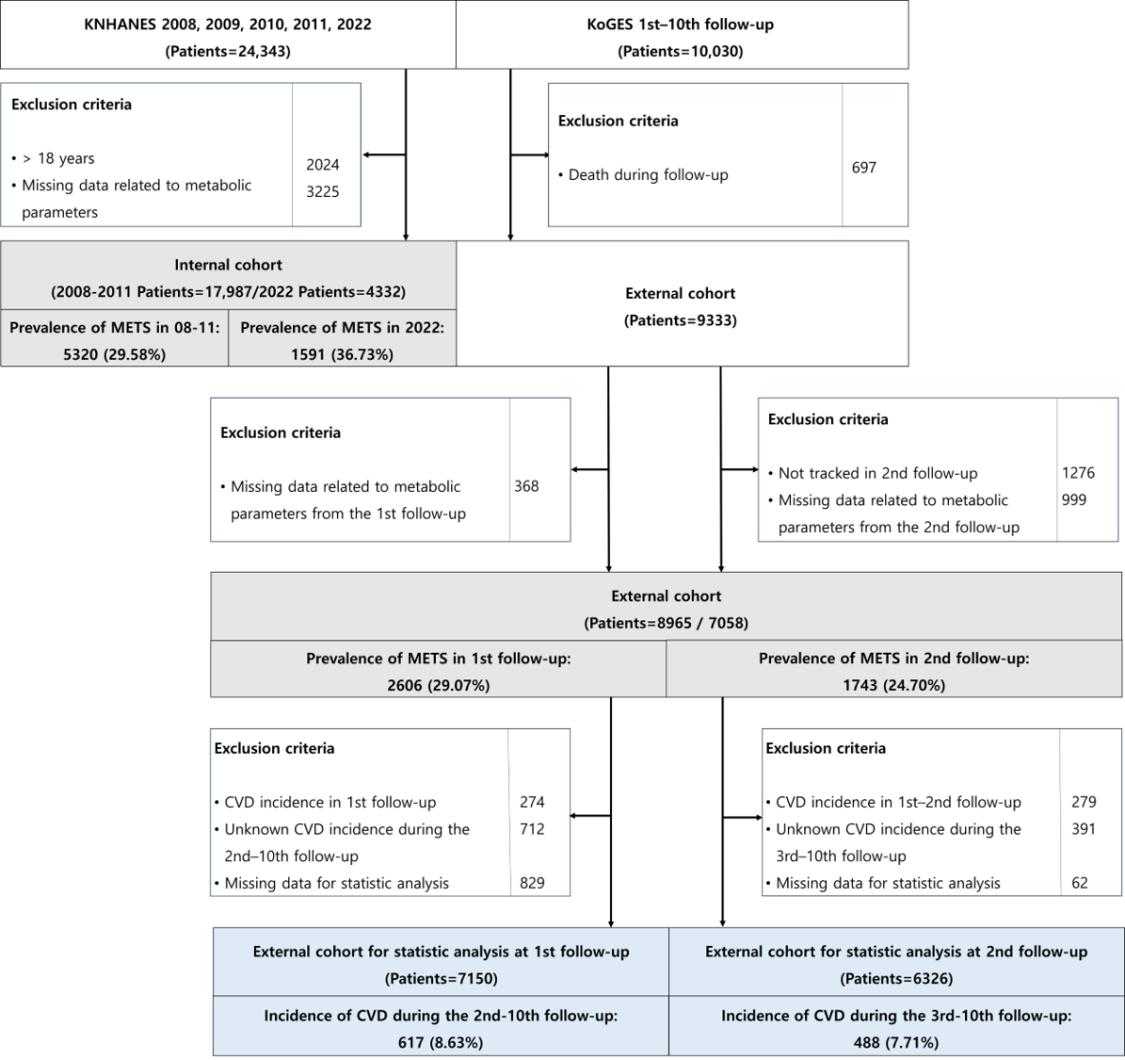
Flowchart for the study cohort formation. The gray background represents the cohort used for model training and validation, while the blue background indicates the cohort used for statistical evaluation of CVD risk. CVD: cardiovascular disease; KNHANES: Korea National Health and Nutrition Examination Survey; KoGES: Korean Genome and Epidemiology Study; METS: metabolic syndrome.

Figure 1 shows a flowchart of the study cohort formation. Of the 24,343 patients included in the 2008-2011 and 2022 KNHANES, 22,319 patients were included in the internal cohort after excluding patients younger than 18 years of age and those with missing data related to metabolic syndrome. Data from the 2008 to 2011 KNHANES, which included DEXA measurements, were used for training, while data from the 2022 KNHANES, which included BIA measurements, were used for internal validation.

Data from the KoGES were used for external validation after excluding patients who died during the follow-up period and those with missing data related to metabolic syndrome. The external validation data included 8965 patients from the first follow-up and 7058 patients from the second follow-up. The prevalence of metabolic syndrome in each cohort was 5320 (29.58%), 1591 (36.73%), 2606 (29.07%), and 1743 (24.70%), respectively.

To evaluate the utility of the model, additional exclusion criteria were applied to the external validation dataset to identify patients with CVD. The analysis was conducted on a longitudinal dataset, where the same cohort of patients was followed across multiple follow-ups. In the first follow-up of the KoGES, 7150 patients were included after excluding those who had developed CVD during the first follow-up, those whose CVD status could not be determined between the second and 10th follow-ups, and those with missing data necessary for statistical analysis. Among these patients, 617 new cases of CVD were identified during the follow-up period (second to 10th follow-up). In the second follow-up of the KoGES, 6326 patients were included after excluding those who developed CVD during the first or second follow-up, those whose CVD status could not be determined between the third and 10th follow-ups, or those with missing data. Among these patients, 488 new cases of CVD were identified during the follow-up period (third to 10th follow-up). The observation period from the first follow-up averaged 5404.20 (SD 1885.38) days, and from the second follow-up averaged 5097.38 (SD 1448.04) days.

### Criteria for Defining Metabolic Syndrome and Study Variables

To define metabolic syndrome, the criteria established by the National Cholesterol Education Program Adult Treatment Panel III were referenced. For abdominal obesity in Korean women, the criteria set by the Korean Society for Obesity in 2006 were used, which defines abdominal obesity as a waist circumference of ≥85 cm. The specific criteria for defining metabolic syndrome in this study were as follows [22,23]: (1) waist circumference: ≥90 cm for men, ≥85 cm for women; (2) triglycerides: ≥150 mg/dL, or currently taking medication for dyslipidemia; (3) HDL cholesterol (HDL-C): <40 mg/dL for men and <50 mg/dL for women or those currently on medication for dyslipidemia; (4) blood pressure: systolic blood pressure of ≥130 mm Hg, diastolic blood pressure of ≥85 mm Hg, or currently taking antihypertensive medication; and (5) fasting glucose: ≥100 mg/dL or currently taking insulin or medication for diabetes. Groups meeting these criteria were defined as those with abdominal obesity, elevated triglycerides, reduced HDL-C, elevated blood pressure, and elevated fasting glucose. Metabolic syndrome was diagnosed if three or more of the five criteria were met. CVD was defined as myocardial infarction, angina pectoris, peripheral artery disease, or stroke based on previous studies [24,25].

Data from the 2008 to 2011 KNHANES cohort measured using DEXA were used for analysis, whereas data from the 2022 KNHANES and the first and second follow-ups of the KoGES cohort measured using BIA were used for validation. Only variables consistently available across all cohorts were considered to ensure that the model could be easily applied without reliance on surveys or additional information. Thus, only sex, age, height, weight, fat mass, and lean mass were used to develop the model, ensuring that it could be used at any time and in any setting.

### Model Development, Validation, and Calibration

We used the KNHANES 2008-2011 data and applied a stratified cross-validation (CV) technique to ensure a similar distribution of metabolic syndrome across five CV folds. These datasets were used for hyperparameter tuning to determine optimal parameters for each model. The KNHANES 2022 data served as internal validation, while the first follow-up of the KoGES was used as external validation 1, and the second follow-up was used as external validation 2. This multistage validation approach aimed to develop a robust model for predicting metabolic syndrome.

In this study, we used six easily obtainable noninvasive data features—age, sex, height, weight, body fat mass, and lean body mass—as input features for predicting the five diagnostic criteria of metabolic syndrome and the presence of metabolic syndrome itself. Model development followed these steps, and using the six features, we directly predicted each of the five diagnostic criteria for metabolic syndrome, as well as metabolic syndrome itself. The best-performing model is selected for each criterion. The best model for each metabolic syndrome diagnostic criterion was used to estimate the probability of meeting that criterion. We compared the area under the receiver operating characteristic curve (AUROC) performance on internal test data, external 1 data, and external 2 data using three different modeling approaches: (1) a model that directly predicts metabolic syndrome using the six features, (2) a model that predicts metabolic syndrome using only the probabilities of each diagnostic criterion derived from their respective best-performing models, and (3) a model that predicts metabolic syndrome using both the probabilities and six features.

To compare the performance of various models for predicting metabolic syndrome and to select the best model, we evaluated several algorithms, including the logistic regression (LR) with various regularization penalties (l1, l2, and elastic net) [26], ensemble-based random forest [27], boosting-based eXtreme gradient boosting [28], multilayer perceptron (MLP) with multiple layers of artificial neural networks [29], and TabNET (TAB) model, which is known for its high performance on tabular datasets [30]. The performances of these models were systematically compared to identify the most robust predictive model for metabolic syndrome.

To preprocess the numerical data, we applied scaling using a robust scaler from Scikit-learn, which effectively handles outliers by removing the median and scaling according to the IQR. To determine the optimal parameters for each model, hyperparameter tuning was performed based on the AUROC performance metric. This tuning process involved specifying a range of hyperparameters for each model and using a 5-fold CV to evaluate performance. After selecting the best parameters for each model, model calibration was applied to improve the probability estimates [31]. We applied two calibration methods (sigmoid and isotonic) in a 5-fold CV setting and evaluated their performance using three metrics—Brier score, expected calibration error, and maximum calibration error. We then chose the calibration method that performed well on at least two of these three metrics.

In addition, we used the Shapley Additive Explanations (SHAP) summary plot to investigate the key factors shaping the predictions of our best-performing model, offering interpretability by quantifying the impact of each feature on its outputs.

### Statistical Analysis and Evaluation of Model Utility

In this study, a series of statistical analyses were conducted to compare the differences between patients with and without metabolic syndrome across various variables and to evaluate the performance of the prediction model. The following analyses were performed.

The 2-tailed *t* tests were used to compare continuous variables between patients with and without metabolic syndrome in the training, internal validation, and external validation datasets. For categorical variables, the chi-square test was used to assess the differences between the two groups.

Next, the relationship between metabolic syndrome and CVD was assessed using Cox proportional hazards regression analysis in the external validation dataset 1 to estimate hazard ratios (HRs) for CVD in patients with metabolic syndrome. The Cox proportional hazards model is widely used in survival analyses to examine the relationship between the timing of an event (in this case, CVD) and one or more predictor variables [32]. This model assumes that the hazard (or risk) of an event is a function of the baseline hazard and a set of covariates that linearly affect the log HR. This approach allows for the adjustment of multiple confounding factors, providing an estimate of how each predictor variable affects the risk of developing CVD while considering the time until an event occurs.

Covariates, such as age, sex, BMI, alcohol consumption, smoking status, and income level, were adjusted for potential confounding effects. Income level was categorized into four groups: <2 million KRW, 2-3 million KRW, 3-4 million KRW, and >4 million KRW (at the time of the study, the average exchange rate was approximately 1220 KRW per US $1). Based on the identified optimal cutoff, HRs for patients with actual metabolic syndrome and those predicted by the model were calculated for both external validation datasets 1 and 2.

In the external validation dataset 1, Cox proportional hazards regression was used to estimate the HRs for CVD in relation to several key variables, including current BMI, body fat percentage, total physical activity, alcohol consumption, and smoking status. All the HRs were adjusted for age, sex, and income level. The same procedure was applied to external validation dataset 2, with the primary variables of interest being baseline metabolic syndrome status, current BMI, change in BMI from baseline, body fat percentage, change in body fat percentage from baseline, current alcohol consumption, and smoking status.

To further assess the practical utility of the model, continuous variables related to physical activity and body composition were examined in relation to CVD risk. Natural cubic splines were applied to model the nonlinear relationships between continuous variables (BMI, body fat percentage, and physical activity) and the risk of developing CVD [33]. Natural splines allow flexible modeling by dividing the range of continuous variables into segments and fitting cubic polynomial functions to each segment. The key advantage of natural splines is their ability to impose linearity beyond boundary knots, ensuring a smoother fit and reducing the risk of overfitting. This makes them particularly useful for capturing complex nonlinear effects that may not be well represented by traditional linear models. All statistical analyses and visualizations, including the application of Cox regression and natural cubic splines, were performed using Python (version 3.11; Python Software Foundation) and SAS (version 9.4; SAS Institute Inc).

## Results

### Baseline Characteristics of Study Population

The baseline characteristics of the variables for each cohort are presented in Table 1. Statistically significant differences were observed between the patients with and without metabolic syndrome for all variables.

**Table 1:** Baseline characteristics.

Cohort and features		Total	Non-METS^a^	METS	*P* value
**Train, n**		17,987	12,667	5320	
	Age (years), mean (SD)	48.92 (16.05)	45.24 (15.63)	57.70 (13.41)	<.001^b^
	Female (person), n (%)	10,215 (56.79)	7388 (58.32)	2827 (53.13)	<.001^b^
	Height (cm), mean (SD)	162.22 (9.21)	162.70 (8.93)	161.08 (9.75)	<.001^b^
	Weight (kg), mean (SD)	62.34 (11.41)	60.20 (10.46)	67.45 (11.96)	<.001^b^
	Body fat (kg), mean (SD)	17.44 (5.71)	16.22 (5.31)	20.34 (5.60)	<.001^b^
	Lean body mass (kg), mean (SD)	44.36 (9.56)	43.45 (9.16)	46.50 (10.14)	<.001^b^
**Internal** **v** **alidation, n**		4332	2741	1591	
	Age (years), mean (SD)	52.46 (16.99)	47.64 (16.85)	60.77 (13.69)	<.001^b^
	Female (person), n (%)	2422 (55.91)	1635 (59.65)	787 (49.47)	<.001^b^
	Height (cm), mean (SD)	163.82 (9.11)	164.29 (8.79)	163.01 (9.60)	<.001^b^
	Weight (kg), mean (SD)	64.95 (13.06)	62.26 (11.80)	69.59 (13.81)	<.001^b^
	Body fat (kg), mean (SD)	19.23 (6.64)	17.53 (5.89)	22.17 (6.85)	<.001^b^
	Lean body mass (kg), mean (SD)	45.70 (9.84)	44.71 (9.46)	47.39 (10.24)	<.001^b^
**External** **v** **alidation 1, n**		8965	6359	2606	
	Age (years), mean (SD)	51.99 (8.84)	50.83 (8.69)	54.80 (8.57)	<.001^b^
	Female (person), n (%)	4742 (52.89)	3227 (50.75)	1515 (58.14)	<.001^b^
	Height (cm), mean (SD)	160.07 (8.65)	160.36 (8.47)	159.36 (9.02)	<.001^b^
	Weight (kg), mean (SD)	63.05 (10.09)	61.12 (9.30)	67.75 (10.39)	<.001^b^
	Body fat (kg), mean (SD)	17.02 (5.54)	15.59 (5.02)	20.50 (5.21)	<.001^b^
	Lean body mass (kg), mean (SD)	46.03 (8.42	45.53 (8.12)	47.25 (8.99	<.001^b^
**External** **validation 2, n**		7058	5315	1743	
	Age (years), mean (SD)	53.65 (8.79)	52.78 (8.66)	56.28 (8.65)	<.001^b^
	Female (person), n (%)	3754 (53.19)	2716 (51.10)	1038 (59.55)	<.001^b^
	Height (cm), mean (SD)	160.08 (9.65)	160.42 (9.79)	159.03 (9.12)	<.001^b^
	Weight (kg), mean (SD)	62.98 (10.03)	61.40 (9.43)	67.79 (10.29)	<.001^b^
	Body fat (kg), mean (SD)	16.66 (5.46)	15.42 (4.96)	20.44 (5.17)	<.001^b^
	Lean body mass (kg), mean (SD)	46.31 (8.44)	45.98 (8.22)	47.35 (9.03)	<.001^b^

^a^METS: metabolic syndrome.

^b^Indicates cases with *P*<.05.

### Model Performance Evaluation

The search ranges for hyperparameter tuning for each model are provided in Multimedia Appendix 1, and the CV results for each model are detailed in Multimedia Appendix 2. Based on the CV results, the LR model achieved the best performance in predicting abdominal obesity with an AUROC of 0.9472. The MLP model was the best-performing model for predicting reduced HDL-C levels, elevated blood pressure, and metabolic syndrome with AUROCs of 0.6982, 0.8129, and 0.8573, respectively. Finally, the TAB model was selected as the best-performing model for predicting elevated triglyceride and fasting glucose levels, with AUROCs of 0.7463 and 0.7542, respectively.

As summarized in Multimedia Appendix 3, we evaluated the calibration performance for each target using three metrics—Brier score, expected calibration error, and maximum calibration error—and selected the method that performed well on at least two of these metrics. The final best model for metabolic syndrome prediction was calibrated using isotonic regression. Additionally, Multimedia Appendix 4 presents the calibration metrics for each target’s chosen calibration method.

Table 2 summarizes the model performance for the internal and external validation datasets. Overall, the models demonstrated better performance on the internal test dataset, with AUROC values ranging from 0.8338 to 0.8447 for metabolic syndrome prediction. The performance differences between the external validation datasets were relatively minor, with AUROCs ranging from 0.8066 to 0.8138 for external dataset 1 and 0.8039 to 0.8123 for external dataset 2. The performance metrics among the three approaches—using only noninvasive features, probability-based predictions derived from each diagnostic criterion, and the combination of both—showed minimal differences. In addition, the performance variation among the five models was not substantial.

**Table 2:** AUROC^a^ performance of models in internal, external validation 1, and external validation 2 datasetsb.

Model and cohort		Target								
		Abdominal obesity	Elevated triglycerides	Reduced HDL-C^c^	Elevated blood pressure	Elevated fasting glucose	METS^d^ (features)	METS (probability)	METS (combination)	
**LR^e^**										
	Internal^f^	0.9556	0.7131	0.7150	0.8164	0.7442	0.8338	0.8447	0.8444	
	External 1^g^	0.9007	0.6832	0.7139	0.7198	0.6508	0.8079	0.8102	0.8097	
	External 2^h^	0.8938	0.6765	0.7075	0.7025	0.6533	0.8054	0.8078	0.8075	
**RF^i^**										
	Internal^f^	0.9510	0.7260	0.7168	0.8115	0.7435	0.8360	0.8393	0.8422	
	External 1^g^	0.8916	0.6960	0.7068	0.7201	0.6550	0.8098	0.8080	0.8118	
	External 2^h^	0.8868	0.6935	0.7033	0.7044	0.6613	0.8065	0.8039	0.8090	
**XGB^j^**										
	Internal^f^	0.9544	0.7287	0.7226	0.8151	0.7450	0.8398	0.8418	0.8412	
	External 1^g^	0.8981	0.6963	0.7043	0.7203	0.6569	0.8113	0.8113	0.8113	
	External 2^h^	0.8923	0.6940	0.7014	0.7051	0.6608	0.8073	0.8093	0.8085	
**MLP^k,l^**										
	Internal^f^	0.9557	0.7414	0.7193	0.8188	0.7467	0.8433	0.8438	0.8437	
	External 1^g^	0.9009	0.7020	0.7143	0.7217	0.6569	0.8138	0.8104	0.8123	
	External 2^h^	0.8939	0.6983	0.7079	0.7075	0.6627	0.8123	0.8096	0.8107	
**TAB^m^**										
	Internal^f^	0.9543	0.7373	0.7181	0.8145	0.7469	0.8406	0.8393	0.8394	
	External 1^g^	0.9030	0.7000	0.7060	0.7211	0.6527	0.8114	0.8066	0.8081	
	External 2^h^	0.8984	0.6984	0.7016	0.7028	0.6614	0.8088	0.8048	0.8067	

^a^AUROC: area under the receiver operating characteristic curve.

^b^The table shows the test data performance for predicting each target using models optimized with the best combination of parameters and calibration. It also presents the test data performance for predicting metabolic syndrome by combining the six features with the predicted probabilities of each diagnostic criterion for metabolic syndrome.

^c^HDL-C: high-density lipoprotein cholesterol.

^d^METS: metabolic syndrome.

^e^LR: logistic regression.

^f^Internal was conducted using the Korea National Health and Nutrition Examination Survey 2022 data.

^g^External 1 was conducted using the first follow-up of the Korean Genome and Epidemiology Study.

^h^External 2 was conducted using the second follow-up of the Korean Genome and Epidemiology Study.

^i^RF: random forest.

^j^XGB: extreme gradient boosting.

^k^MLP: multilayer perceptron.

^l^Represents the final selected model in the cross-validation process.

^m^TAB: TabNET.

In the internal dataset, the LR model achieved the highest AUROC of 0.8447 when predicting metabolic syndrome using the probabilities from the best-performing models for each criterion as a feature. For external datasets, the MLP model demonstrated the best performance in noninvasive feature-based metabolic syndrome prediction, achieving AUROCs of 0.8138 and 0.8123 in external datasets 1 and 2, respectively.

To further examine the key factors influencing predictions, we evaluated the feature importance of this best-performing model in the external validation cohorts using SHAP. The SHAP analysis identified sex as the most critical feature, followed by lean mass and body fat mass. Given the prominent role of sex, we conducted a subgroup analysis stratified by sex to investigate both model performance and feature contribution differences. These subgroup results, including performance metrics and SHAP-based interpretations, are presented in Multimedia Appendices 5 and 6.

### Model Utility Evaluation with CVD Risk Assessment

Multimedia Appendix 7 illustrates how precision, recall, and *F*_1_-score vary across different threshold values for predicting metabolic syndrome in the internal validation dataset. We clarify that we selected the threshold by identifying the point at which the *F*_1_-score was maximized (0.32), thereby balancing precision and recall. In addition, after selecting this threshold, we applied the model to external datasets to predict metabolic syndrome and subsequently assessed the associated risk of CVD.

**Figure 2:**
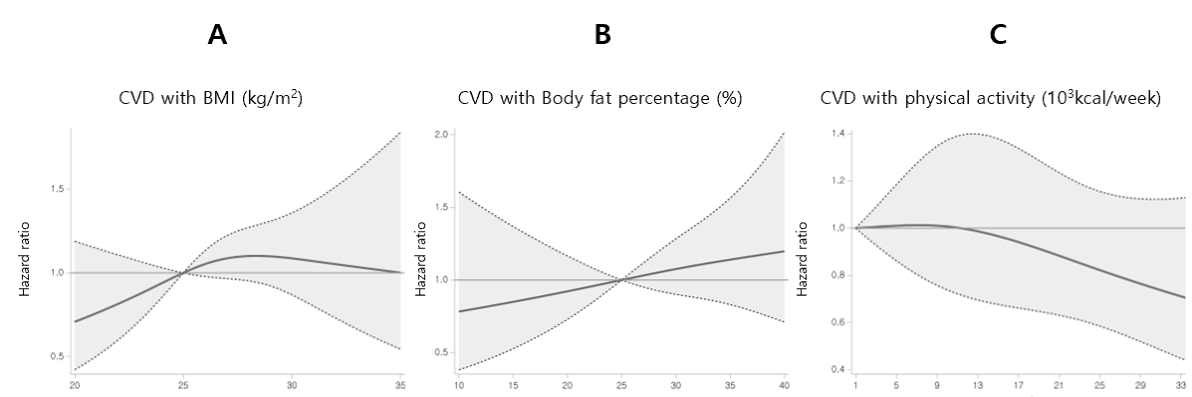
CVD HRs by continuous variables. Solid lines represent adjusted HRs and shaded regions indicate 95% CIs from restricted cubic spline regression. The CVD HR was calculated for patients predicted to have metabolic syndrome using this model. The HRs were adjusted for sex, age, alcohol consumption, smoking status, and income level. (A) The HR based on BMI, with a reference value of 25. (B) The HR based on body fat percentage, with a reference value of 25. (C) The HR based on physical activity, with the reference value set at 1. CVD: cardiovascular disease; HR: hazard ratio.

**Figure 3:**
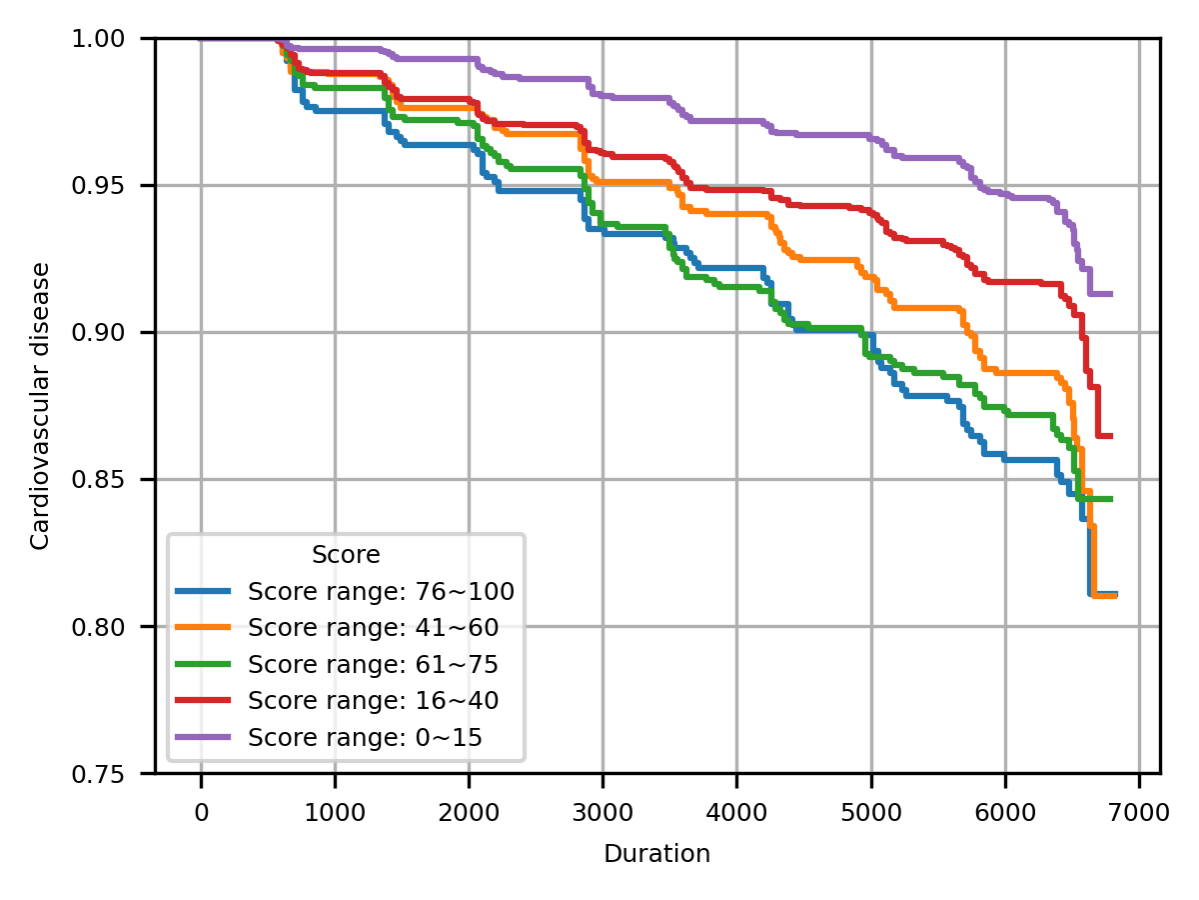
Kaplan-Meier survival curves for cardiovascular disease according to metabolic syndrome risk groups.

Table 3 shows the baseline characteristics of the variables used in the statistical analyses. Additionally, Table 4 shows the HRs calculated using Cox proportional hazard models for each cohort. Figure 2 shows the CVD HRs based on continuous variables such as BMI, body fat mass percentage, and total physical activity for patients with metabolic syndrome and those predicted to have metabolic syndrome in the external validation cohort 1. The x-axis of each graph represents the respective continuous variables, whereas the y-axis shows the HRs, indicating the impact of the variables on CVD occurrence. The solid line represents the estimated HRs and the dashed lines indicate the 95% CIs. The first column shows the relationship between the BMI and CVD risk. When BMI was <25, the CVD risk tended to decrease. The second column represents the relationship between body fat percentage and CVD risk, where an increase in body fat percentage tended to increase CVD risk. The third column illustrates the relationship between physical activity and CVD risk. As physical activity increased, CVD risk tended to decrease, especially in the predicted metabolic syndrome group, in which burning more than approximately 9000 kcal per week correlated with decreased CVD risk. This suggests that engaging in exercise beyond routine physical activity can further reduce the risk of CVD. The statistical results for patients with actual metabolic syndrome are shown in Multimedia Appendices 8-10. Finally, Figure 3 presents the Kaplan-Meier survival curves for CVD according to the different metabolic syndrome risk probability groups predicted by the model. As shown in Multimedia Appendices 11-13, the differences in the survival curves across most score intervals were statistically significant.

**Table 3:** Statistics for the statistical analysis cohort.

Cohort and features				Total		Non-CVD^a^		CVD		*P* value	
**Predicted METS^b^ (external validation 1), n**				3698		3288		410			
	Incidence of METS at the second follow-up^c^, n (%)			1328 (35.91)		1167 (35.49)		161 (39.27)		.03^d^	
	Age (years), mean (SD)			54.27 (8.53)		54.00 (8.58)		56.45 (7.77)		<.001^d^	
	Female (person), n (%)			1961 (53.03)		1746 (53.10)		215 (52.44)		.84	
	BMI (kg/m^2^), mean (SD)			26.66 (2.45)		26.68 (2.46)		26.48 (2.35)		.13	
	Body fat (%), mean (SD)			30.04 (6.09)		30.04 (6.10)		30.00 (6.02)		.89	
	Alcohol (person), n (%)			1700 (45.97)		1524 (46.35)		176 (42.93)		.21	
	Smoke (person), n (%)			802 (21.69)		702 (21.35)		100 (24.39)		.18	
	PA^e^ (kcal/week), mean (SD)			11,797 (7306)		11,797 (7321)		11,797 (7192)		≥.99	
	**Income (million KRW)^f^, n (%)**										.52
		2	2458 (66.47)		2177 (66.21)		281 (68.54)				
		2, 4	951 (25.72)		849 (25.82)		102 (24.88)				
		4	289 (7.82)		262 (7.97)		27 (6.59)				
**Predicted METS (external validation 2), n**				3368		3049		319			
	History of METS at the first follow-up, n (%)			1379 (40.94)		1214 (39.82)		165 (51.72)		<.001^d^	
	Age (years), mean (SD)			55.54 (8.40)		55.28 (8.42)		57.97 (7.80)		<.001^d^	
	Female (person), n (%)			1833 (54.42)		1660 (54.44)		173 (54.23)		.99	
	BMI (kg/m^2^), mean (SD)			26.61 (4.12)		26.64 (4.27)		26.35 (2.20)		.23	
	Change in BMI (kg/m^2^), mean (SD)			0.23 (3.60)		0.24 (3.77)		0.12 (0.98		.56	
	Body fat, %			29.58±6.12		29.58±6.15		29.60 (5.87)		.97	
	Change in body fat (%), mean (SD)			–0.01 (3.10)		0.00 (3.12)		–0.12 (2.87)		.53	
	**Alcohol (person), n (%)**										.39
		Nonalcoholic	1558 (46.26)		1403 (46.02)		155 (48.59)				
		Abstinent	188 (5.58)		167 (5.48)		21 (6.58)				
		Current alcohol use	1622 (48.16)		1479 (48.51)		143 (44.83)				
	**Smoke (person), n (%)**										.11
		Nonsmoker	2562 (76.07)		2328 (76.35)		234 (73.35)				
		Former smoker	169 (5.02)		157 (5.15)		12 (3.76)				
		Current smoker	637 (18.91)		564 (18.50)		73 (22.88)				
	**Income** **(** **million KRW), n (%)**										.03^d^
		2	2158 (64.07)		1932 (63.37)		226 (70.85)				
		2, 4	908 (26.96)		836 (27.42)		72 (22.57)				
		4	302 (8.97)		281 (9.22)		21 (6.58)				

^a^CVD: cardiovascular disease.

^b^METS: metabolic syndrome.

^c^409 individuals with missing metabolic syndrome data at the 2nd follow-up were excluded.

^d^Indicates cases with *P*<.05.

^e^PA: physical activity.

^f^At the time of the study (2001-2004), the average exchange rate was approximately 1220 KRW per US $1.

**Table 4:** HRs^a^ for cardiovascular disease by characteristics.

Cohort and features			HR (95% CI)			*P* value		aHR^b^ (95% CI)	*P* value	
**Predicted METS^c^ (external validation 1)**										
	**BMI (kg/m^2^)**									
		<23		1.00 (reference)	—^d^		1.00 (reference)			—
		≥23, <25		1.04 (0.61-1.76)	.90		1.23 (0.72-2.10)			.46
		≥25		0.83 (0.50-1.37)	.47		1.25 (0.73-2.12)			.42
	**Body fat (%)**									
		<Q25		1.00 (reference)	—		1.00 (reference)			—
		≥Q25, <Q75		1.02 (0.69-1.50)	.93		1.08 (0.73-1.61)			.69
		≥Q75		1.07 (0.72-1.59)	.74		1.32 (0.80-2.17)			.27
	**PA^e^ (103 kcal/week)**									
		<Q25		1.00 (reference)	—		1.00 (reference)			—
		≥Q25, <Q75		1.03 (0.80-1.33)	.80		1.09 (0.84-1.41)			.51
		≥Q75		0.99 (0.75-1.30)	.93		0.93 (0.70-1.24)			.64
	**Drink**									
		No		1.00 (reference)	—		1.00 (reference)			—
		Yes		0.84 (0.69-1.03)	.09		0.86 (0.69-1.09)			.22
	**Smoke**									
		No		1.00 (reference)	—		1.00 (reference)			—
		Yes		1.20 (0.96-1.51)	.11		1.44 (1.10–1.87)			.008^f^
**Predicted METS (external validation 2)**										
	**History of METS at the first follow-up**									
		No		1.00 (reference)	—		1.00 (reference)			—
		Yes		1.61 (1.30-2.01)	<.001^f^		1.52 (1.21-1.91)			<.001^f^
	**BMI (kg/m^2^)**									
		<23		1.00 (reference)	—		1.00 (reference)			—
		≥23, <25		1.55 (0.75-3.21)	.23		1.78 (0.85-3.75)			.13
		≥25		1.26 (0.62-2.56)	.52		1.78 (0.84-3.78)			.13
	**Change in BMI (kg/m^2^)**									
		<Q25		1.00 (reference)	—		1.00 (reference)			—
		≥Q25, <Q75		1.01 (0.76-1.34)	.96		1.04 (0.77-1.41)			.80
		≥Q75		1.05 (0.77-1.42)	.76		1.18 (0.83-1.66)			.36
	**Body fat (%)**									
		<Q25		1.00 (reference)	—		1.00 (reference)			—
		≥Q25, <Q75		0.94 (0.61-1.45)	.78		0.99 (0.63-1.55)			.96
		≥Q75		0.91 (0.59-1.43)	.69		0.92 (0.53-1.62)			.78
	**Change in body fat (%)**									
		<Q25		1.00 (reference)	—		1.00 (reference)			—
		≥Q25, <Q75		0.92 (0.69-1.22)	.55		0.96 (0.71-1.30)			.80
		≥Q75		0.90 (0.65-1.23)	.49		0.86 (0.60-1.22)			.40
	**Drink**									
		Nonalcoholic		1.00 (reference)	—		1.00 (reference)			—
		Abstinent		1.08 (0.68-1.70)	.74		1.14 (0.72-1.80)			.59
		Current alcohol use		0.88 (0.70-1.10)	.26		0.92 (0.71-1.20)			.55
	**Smoke**									
		Nonsmoker		1.00 (reference)	—		1.00 (reference)			—
		Former smoker		0.77 (0.43-1.38)	.38		0.87 (0.47-1.60)			.65
		Current smoker		1.33 (1.02-1.72)	.04^f^		1.52 (1.11-2.10)			.01^f^

^a^HR: hazard ratio.

^b^aHR: adjusted hazard ratios; corrected for factors such as sex and age.

^c^METS: metabolic syndrome.

^d^Not applicable.

^e^PA: physical activity.

^f^Cases with *P*<.05.

## Discussion

### Principal Findings

We developed a high-performing metabolic syndrome prediction model using only noninvasive data, probability estimates from models that predicted each diagnostic criterion, or a combination of both, achieving AUROC values ranging from 0.8039 to 0.8447 across internal, external 1, and external 2 validation datasets. Patients predicted to have metabolic syndrome by the final model exhibited high CVD risk similar to actual patients with metabolic syndrome. Furthermore, the model demonstrated practical utility by suggesting specific and actionable management strategies, such as increasing physical activity, to lower the risk of CVD development in cases predicted as metabolic syndrome. This approach, which allows patients to easily check their current health status, raises awareness about their health, and provides appropriate management through web- or app-based platforms, can be effective in helping patients lead healthier lives [34-36].

By using noninvasive body composition data such as anthropometric measurements, health care providers can perform more practical and scalable screenings for metabolic syndrome [37], thereby facilitating timely interventions to mitigate the risks of CVD and other related chronic conditions. From the perspective of body composition measurements, DEXA is considered the gold standard method for analyzing body composition due to its precision in measuring fat and lean body mass [38]. However, their limited accessibility in routine clinical settings highlights the importance of alternative methods. In this context, BIA has gained traction as a more accessible and cost-effective tool despite its lower accuracy compared to DEXA. BIA’s practicality makes it a valuable asset in community health programs, where rapid and widespread screening is essential [39]. With the increasing availability of home-based body composition scales such as bioelectrical impedance devices, individuals can conveniently monitor key noninvasive metrics such as body fat percentage and muscle mass [40]. This makes it feasible to predict metabolic disorders and CVD risk outside the clinical environment, thus supporting broader health management strategies in daily life [41].

### Comparison to Prior Work

Our model demonstrated comparable or superior performance to those of previous studies in predicting metabolic syndrome using noninvasive data. However, these studies typically included features such as blood pressure and waist circumference, which are part of the current labeling criteria for metabolic syndrome [12], incorporated data with reliability concerns, such as dietary or physical activity questionnaires [42,43], or used genome-related data, such as genome-wide polygenic risk scores, which are not easily accessible in routine clinical or community health settings in their model training [44].

Another important contribution of this study is the evaluation of the utility of the model in predicting long-term CVD risk, which is a critical complication of metabolic syndrome. Using Cox proportional hazards regression analysis, the study confirmed that patients classified as having metabolic syndrome based on the model’s predictions had a significantly higher risk of developing CVD than patients without metabolic syndrome, with an estimated HR of 1.51. This finding highlights the broader applicability of the predictive model for identifying individuals at a higher risk of CVD, thus enabling early intervention and preventive care strategies. Although several studies have successfully predicted metabolic syndrome using noninvasive data, to the best of our knowledge, this is the first study to offer additional clinical interpretations such as the risk of CVD [14,45].

### Limitations

Despite the high performance of our METS prediction model for metabolic syndrome, several limitations should be acknowledged. First, the BIA data used for the external validation can vary significantly depending on the device used. In contrast, the training data relied on DEXA, which is the gold standard method for analyzing body composition. The lower performance observed with BIA compared with DEXA highlights potential areas for improvement as BIA technology continues to evolve. Second, although this study used a large multicenter cohort, the population was predominantly Korean, which limits the generalizability of the findings across different ethnicities. Future studies should include diverse populations. Third, among the five diagnostic criteria of metabolic syndrome, we observed lower predictive performance for elevated triglycerides, reduced HDL-C, elevated blood pressure, and elevated fasting glucose compared with abdominal obesity. This suggests that body composition data alone may be insufficient to fully capture the more complex metabolic processes underlying these nonobesity components. Fourth, in external validation 2, the proportion of individuals with a history of metabolic syndrome differed considerably between patients with predicted and actual metabolic syndrome. While we aimed to use minimal features that could be easily captured by BIA, the inclusion of additional variables such as past medical history or simple survey-based characteristics such as smoking and alcohol consumption could enhance the model’s predictive power in the future. Finally, we only assessed the utility of our metabolic syndrome model in predicting CVD risk. Further studies should explore its applicability to other health outcomes and management indicators to provide a more comprehensive evaluation of the utility of the model.

### Clinical Implications and Future Directions

The findings of this study align with previous research highlighting the underdiagnosis of metabolic syndrome in primary care settings. A previous cross-sectional study conducted in Australian physiotherapy private practices found that 37% of participants had metabolic syndrome, yet none were aware of their condition [46]. This underscores a critical gap in routine metabolic risk screening, even among individuals already engaging with the health care system.

This study further addresses this gap by providing a noninvasive, accessible predictive model that enables early detection of metabolic syndrome without requiring laboratory tests. By integrating this model into primary care, public health programs, and digital health platforms, health care providers can efficiently identify high-risk individuals and implement timely interventions. Moreover, given that many individuals with metabolic syndrome remain undiagnosed until CVD arises, this model has the potential to prevent disease progression by facilitating proactive risk management and personalized lifestyle modifications.

Beyond clinical practice, the model’s scalability extends to large-scale screening initiatives and community health programs, where noninvasive risk assessment can drive cost-effective, widespread prevention efforts. As a previous study has shown that lifestyle interventions can effectively improve cardiometabolic health in individuals with metabolic syndrome [47], integrating predictive tools into routine health care could empower both healthy controls and patients with metabolic syndrome to take a more active role in managing their health. Ultimately, by bridging the gap between metabolic syndrome screening and long-term CVD risk prediction, this model presents a practical solution for reducing the burden of cardiometabolic diseases at both individual and population levels.

Future research should focus on incorporating more diverse populations and exploring the utility of this model in predicting health outcomes other than CVD. Additionally, the utility of this model should be further evaluated for predicting health outcomes beyond CVD, including diabetes, nonalcoholic fatty liver disease, and other metabolic disorders. Furthermore, implementation studies are needed to assess the real-world impact of deploying this model in various health care settings, particularly in resource-limited environments where invasive testing may not be feasible. Finally, exploring how this model can be integrated into existing digital health platforms would facilitate its adoption in routine clinical practice.

### Conclusions

This study successfully developed a predictive model for metabolic syndrome using noninvasive body composition data, demonstrating strong performance in both internal and external validation cohorts. The use of both DEXA and BIA data for training and validation improved the generalizability of the model across different clinical settings, making it suitable for widespread use where DEXA is not available. Our model outperformed previous studies by relying solely on noninvasive features and offering additional clinical insights, such as the risk of developing CVD. Clinically, this model offers a practical solution for early detection and intervention of metabolic syndrome in various health care settings, particularly in primary care or community health programs where invasive testing may not be feasible. By enabling proactive health management, it can contribute to reducing the long-term burden of cardiometabolic diseases, ultimately improving patient outcomes and quality of life. However, the limitations include variability in BIA data, lack of ethnic diversity in the cohort, and exclusion of potentially useful features, such as medical history and lifestyle factors. Future research should focus on incorporating more diverse populations and exploring the utility of this model in predicting health outcomes other than CVD.

## Appendix

Multimedia Appendix 1 Results of hyperparameter tuning.

Multimedia Appendix 2 Cross-validation performance of metabolic syndrome prediction models.

Multimedia Appendix 3 Calibration performance of each model, calibration method, and target.

Multimedia Appendix 4 Calibration performance of final calibrated models in internal and external validation sets.

Multimedia Appendix 5 Sex-stratified performance of the best-performing model for metabolic syndrome in internal and external validation datasets.

Multimedia Appendix 6 Shapley Additive Explanations value of the model. The Shapley Additive Explanations values pertain to the final developed model, where (A) represents the Shapley Additive Explanations value results for the entire cohort, and (B) and (C) indicate the Shapley Additive Explanations value results for males and females, respectively.

Multimedia Appendix 7 Performance variation of the model based on different thresholds. The results show precision, recall, and F1-score evaluated in the internal cohort by increasing the threshold from 0 to 1 in increments of 0.01.

Multimedia Appendix 8 Statistics for the statistical analysis cohort.

Multimedia Appendix 9 Hazard ratios for cardiovascular disease by characteristics.

Multimedia Appendix 10 Cardiovascular disease hazard ratios by continuous variables.

Multimedia Appendix 11 Prevalence of metabolic syndrome and incidence of cardiovascular disease across metabolic syndrome risk groups.

Multimedia Appendix 12 Adjusted hazard ratios for cardiovascular disease based on metabolic syndrome status and the model's predicted probability in external cohort 1. The red line represents the adjusted hazard ratios for individuals with metabolic syndrome. Dotted lines indicate the 95% CIs.

Multimedia Appendix 13 Statistical differences between metabolic syndrome risk groups.

## Abbreviations

AUROC: area under the receiver operating characteristic curve
BIA: bioelectrical impedance analysis
CV: cross-validation
CVD: cardiovascular disease
DEXA: dual-energy x-ray absorptiometry
HDL: high-density lipoprotein
HDL-C: high-density lipoprotein cholesterol
HR: hazard ratio
KNHANES: Korea National Health and Nutrition Examination Survey
KoGES: Korean Genome and Epidemiology Study
LR: logistic regression
MLP: multilayer perceptron
SHAP: Shapley Additive Explanations

## Data Availability

The Korea National Health and Nutrition Examination Survey data are publicly available and were used in this study. The Korean Genome and Epidemiology Study data contain sensitive personal information and are not publicly available; access is subject to institutional review and approval. Additionally, the machine learning–based predictive system developed in this study is accessible as a web-based platform. Full details on data sources and platform access are provided in the reference list [48-50].
