# Supplementary material for: Development of a Predictive Model for Metabolic Syndrome Using Noninvasive Data and its Cardiovascular Disease Risk Assessments: Multicohort Validation Study
Source: J Med Internet Res. 2025 May 2;27:e67525. doi: 10.2196/67525 (PMC12084770; doi:10.2196/67525)
Supplement: Multimedia Appendix 1 [file jmir_v27i1e67525_app1.docx]

| Model | Hyperparameter | Tuning Range |
| --- | --- | --- |
| LR | Penalty | ['l1', 'l2', 'elasticnet', None] |
|  | C | [0.01, 0.1, 1, 10, 100] |
|  | Solver | ['liblinear', 'saga', 'newton-cg', 'lbfgs', 'sag'] |
|  | Max Iterations | [100, 200, 300, 500] |
|  | Tolerance (tol) | [1e-4, 1e-3, 1e-2] |
|  | L1 Ratio | [0.5] |
| RF | Number of Estimators | [100, 300, 500] |
|  | Max Depth | [None, 5, 6, 7, 8, 9, 10] |
|  | Min Samples Split | [2, 3, 4, 5] |
|  | Min Samples Leaf | [1, 2, 3, 5] |
|  | Bootstrap | [True, False] |
|  | Class Weight | [None, 'balanced'] |
| XGB | Number of Estimators | [100, 200, 500] |
|  | Max Depth | [3, 5, 7, 10] |
|  | Learning Rate | [0.01, 0.05, 0.1, 0.2] |
|  | Subsample | [0.5, 0.7, 1.0] |
|  | Colsample by Tree | [0.5, 0.7, 1.0] |
|  | Gamma | [0, 1, 5] |
|  | Regularization Alpha | [0, 0.1, 0.5] |
|  | Regularization Lambda | [0, 0.1, 0.5] |
| MLP | Hidden Layer Sizes | [(50,), (100,), (50, 50)] |
|  | Activation | ['relu', 'tanh'] |
|  | Solver | ['adam', 'sgd'] |
|  | Alpha | [0.0001, 0.001] |
|  | Batch Size | [64, 128] |
|  | Learning Rate | ['constant', 'adaptive'] |
|  | Max Iterations | [200, 400] |
| TAB | Number of Steps | [2, 3] |
|  | Gamma | [0.8, 1, 1.2] |
|  | Number of Independents | [2, 3, 4] |
|  | Number of Shared | [2, 3, 4] |
|  | Momentum | [0.01, 0.02, 0.03] |
|  | Optimizer Function | [torch.optim.Adam] |
|  | Optimizer Parameters | [dict(lr=2e-2)] |
|  | Scheduler Parameters | [{'step_size': 50, 'gamma': 0.9}] |
|  | Scheduler Function | [torch.optim.lr_scheduler.StepLR] |
|  | Mask Type | ['entmax'] |
| **Abbreviations**: LR, logistic regression; RF, random forest; XGB, extreme gradient boosting; MLP, multi-layer perceptron; TAB, tabnet.  ***Notes***: The parameter search ranges shown are used to identify the best parameters for each model based on AUROC performance in five-fold cross-validation. | | |
