# Supplementary material for: Development of a Predictive Model for Metabolic Syndrome Using Noninvasive Data and its Cardiovascular Disease Risk Assessments: Multicohort Validation Study
Source: J Med Internet Res. 2025 May 2;27:e67525. doi: 10.2196/67525 (PMC12084770; doi:10.2196/67525)
Supplement: Multimedia Appendix 2 [file jmir_v27i1e67525_app2.docx]

| Variable^a^ | | Target | | | | | |
| --- | --- | --- | --- | --- | --- | --- | --- |
|  |  | Abdominal Obesity | Elevated Triglycerides | Reduced  HDL-C | Elevated Blood Pressure | Elevated Fasting Glucose | Metabolic Syndrome |
| LR | Accuracy | 0.8865 | 0.6954 | 0.6447 | 0.7390 | 0.7325 | 0.7906 |
|  | F1 Score | 0.8848 | 0.6811 | 0.6426 | 0.7376 | 0.6952 | 0.7828 |
|  | AUROC | **0.9472** | 0.7234 | 0.6951 | 0.8073 | 0.7457 | 0.8497 |
|  | AUPRC | 0.8698 | 0.4869 | 0.6420 | 0.7091 | 0.4938 | 0.6793 |
| RF | Accuracy | 0.8818 | 0.7037 | 0.6438 | 0.7373 | 0.7380 | 0.7933 |
|  | F1 Score | 0.8807 | 0.6990 | 0.6420 | 0.7373 | 0.7207 | 0.7887 |
|  | AUROC | 0.9412 | 0.7430 | 0.6957 | 0.8104 | 0.7517 | 0.8536 |
|  | AUPRC | 0.8607 | 0.5233 | 0.6461 | 0.7146 | 0.5055 | 0.6898 |
| XGB | Accuracy | 0.8843 | 0.7081 | 0.6430 | 0.7388 | 0.7367 | 0.7950 |
|  | F1 Score | 0.8838 | 0.6954 | 0.6417 | 0.7387 | 0.7087 | 0.7922 |
|  | AUROC | 0.9459 | 0.7449 | 0.6968 | 0.8121 | 0.7522 | 0.8558 |
|  | AUPRC | 0.8669 | 0.5248 | 0.6479 | 0.7178 | 0.5076 | 0.6953 |
| MLP | Accuracy | 0.8851 | 0.7076 | 0.6461 | 0.7400 | 0.7392 | 0.7971 |
|  | F1 Score | 0.8831 | 0.6893 | 0.6454 | 0.7401 | 0.7060 | 0.7923 |
|  | AUROC | 0.9472 | 0.7454 | **0.6982** | **0.8129** | 0.7527 | **0.8573** |
|  | AUPRC | 0.8703 | 0.5288 | 0.6501 | 0.7219 | 0.5052 | 0.6969 |
| TAB | Accuracy | 0.8844 | 0.7091 | 0.6428 | 0.7378 | 0.7381 | 0.7938 |
|  | F1 Score | 0.8828 | 0.6913 | 0.6413 | 0.7377 | 0.7049 | 0.7877 |
|  | AUROC | 0.9466 | **0.7463** | 0.6959 | 0.8127 | **0.7542** | 0.8566 |
|  | AUPRC | 0.8695 | 0.5285 | 0.6466 | 0.7191 | 0.5050 | 0.6941 |
| **Abbreviations**: LR, logistic regression; RF, random forest; XGB, extreme gradient boosting; MLP, multi-layer perceptron; TAB, tabnet; AUROC, area under the receiver operating characteristic curve; AUPRC, area under the precision-recall curve.  ***Notes***: The AUROC best performance for each target was indicated in bold. ^a^ Each performance metric represents the average performance obtained from five-fold cross-validation using the best combination of parameters and calibration for each model type. | | | | | | | |
