# Supplementary material for: Development of a Predictive Model for Metabolic Syndrome Using Noninvasive Data and its Cardiovascular Disease Risk Assessments: Multicohort Validation Study
Source: J Med Internet Res. 2025 May 2;27:e67525. doi: 10.2196/67525 (PMC12084770; doi:10.2196/67525)
Supplement: Multimedia Appendix 3 [file jmir_v27i1e67525_app3.docx]

| Variable | | | Target | | | | | | | |
| --- | --- | --- | --- | --- | --- | --- | --- | --- | --- | --- |
| Model | Calibration Methods | Calibration Evaluation | Abdominal Obesity | Elevated Triglycerides | Reduced  HDL-C | Elevated Blood Pressure | Elevated Fasting Glucose | METS  (Features Only) | METS  (Probability Only) | METS  (Combination) |
| LR | Sigmoid | Brier score | 0.0802 | 0.1896 | 0.2209 | 0.1750* | 0.1724 | 0.1416 | 0.1368 | 0.1368 |
|  |  | ECE | 0.0266* | 0.1438 | 0.0478* | 0.0312 | 0.0504* | 0.0311* | 0.0296 | 0.0288 |
|  |  | MCE | 0.0747* | 0.5751 | 0.1756* | 0.0773* | 0.1733* | 0.0823* | 0.0661* | 0.0621* |
|  | Isotonic | Brier score | 0.0802 | 0.1870* | 0.2208* | 0.1751 | 0.1724 | 0.1413* | 0.1364* | 0.1366* |
|  |  | ECE | 0.0294 | 0.0659* | 0.0546 | 0.0285* | 0.0540 | 0.0393 | 0.0207* | 0.0233* |
|  |  | MCE | 0.0824 | 0.3719* | 0.2993 | 0.1112 | 0.2123 | 0.2511 | 0.0902 | 0.0981 |
| RF | Sigmoid | Brier score | 0.0876 | 0.1846* | 0.2211* | 0.1751 | 0.1728 | 0.1427 | 0.1412 | 0.1389 |
|  |  | ECE | 0.0910 | 0.0533* | 0.0337* | 0.0387* | 0.0611* | 0.0625 | 0.0576 | 0.0458 |
|  |  | MCE | 0.2027 | 0.1144* | 0.0854* | 0.0800* | 0.1697* | 0.1280 | 0.1273 | 0.0982 |
|  | Isotonic | Brier score | 0.0843* | 0.1849 | 0.2213 | 0.1747* | 0.1721* | 0.1409* | 0.1390* | 0.1373* |
|  |  | ECE | 0.0559* | 0.0985 | 0.0582 | 0.0391 | 0.0785 | 0.0439* | 0.0431* | 0.0286* |
|  |  | MCE | 0.1523* | 0.3414 | 0.2544 | 0.1030 | 0.2557 | 0.1210* | 0.1057* | 0.0895* |
| XGB | Sigmoid | Brier score | 0.0840 | 0.1832 | 0.2201* | 0.1736 | 0.1714 | 0.1408 | 0.1383 | 0.1386 |
|  |  | ECE | 0.0705 | 0.0696* | 0.0277* | 0.0330 | 0.0529* | 0.0492 | 0.0438 | 0.0465 |
|  |  | MCE | 0.1543 | 0.3321* | 0.0774* | 0.0769 | 0.1731* | 0.0996 | 0.0950 | 0.0954* |
|  | Isotonic | Brier score | 0.0813* | 0.1829* | 0.2203 | 0.1730* | 0.1708* | 0.1389* | 0.1368* | 0.1371* |
|  |  | ECE | 0.0376* | 0.0854 | 0.0486 | 0.0264* | 0.0590 | 0.0335* | 0.0284* | 0.0289* |
|  |  | MCE | 0.1465* | 0.4528 | 0.2232 | 0.0693* | 0.2227 | 0.0777* | 0.0850* | 0.1066 |
| MLP | Sigmoid | Brier score | 0.0827 | 0.1825 | 0.2197* | 0.1734 | 0.1710 | 0.1396 | 0.1382 | 0.1384 |
|  |  | ECE | 0.0609 | 0.0560* | 0.0560* | 0.0347 | 0.0514 | 0.0389 | 0.0464 | 0.0395 |
|  |  | MCE | 0.1530 | 0.2359* | 0.2859* | 0.0723 | 0.1572* | 0.0788* | 0.1072 | 0.0947 |
|  | Isotonic | Brier score | 0.0802* | 0.1818* | 0.2199 | 0.1727* | 0.1703* | 0.1379* | 0.1365* | 0.1367* |
|  |  | ECE | 0.0300* | 0.0684 | 0.0889 | 0.0247* | 0.0415* | 0.0285* | 0.0227* | 0.0242* |
|  |  | MCE | 0.0680* | 0.3600 | 0.5222 | 0.0621* | 0.1876 | 0.0975 | 0.0749* | 0.0700* |
| TAB | Sigmoid | Brier score | 0.0849 | 0.1850 | 0.2231* | 0.1760 | 0.1727 | 0.1411* | 0.1396 | 0.1405 |
|  |  | ECE | 0.0696 | 0.0656 | 0.0384* | 0.0369 | 0.0560* | 0.0470 | 0.0460 | 0.0500 |
|  |  | MCE | 0.1797 | 0.1976 | 0.1175* | 0.0764* | 0.1640* | 0.0912* | 0.1096 | 0.0930* |
|  | Isotonic | Brier score | 0.0821* | 0.1838* | 0.2236 | 0.1753* | 0.1719* | 0.1394 | 0.1378* | 0.1387* |
|  |  | ECE | 0.0282* | 0.0521* | 0.0696 | 0.0275* | 0.0689 | 0.0300* | 0.0274* | 0.0311* |
|  |  | MCE | 0.0803* | 0.1592* | 0.3068 | 0.0921 | 0.3611 | 0.0973 | 0.0890* | 0.0966 |
| **Abbreviations**: LR, logistic regression; RF, random forest; XGB, extreme gradient boosting; MLP, multi-layer perceptron; TAB, tabnet; METS, metabolic syndrome; HDL-C, high-density lipoprotein cholesterol; ECE, expected calibration error; MCE, maximum calibration error.  ***Notes***: Brier score, ECE, and MCE were measured for each combination of model, calibration method, and target. Lower values indicate better calibration for each metric. Asterisks (*) indicate superior performance compared to the other calibration method (Sigmoid vs. Isotonic) for the same model, target, and metric. | | | | | | | | | | |
