# Supplementary material for: Development of a Predictive Model for Metabolic Syndrome Using Noninvasive Data and its Cardiovascular Disease Risk Assessments: Multicohort Validation Study
Source: J Med Internet Res. 2025 May 2;27:e67525. doi: 10.2196/67525 (PMC12084770; doi:10.2196/67525)
Supplement: Multimedia Appendix 4 [file jmir_v27i1e67525_app4.docx]

| Variable | | | Target | | | | | | | |
| --- | --- | --- | --- | --- | --- | --- | --- | --- | --- | --- |
| Model | Validation Cohort | Calibration Evaluation | Abdominal Obesity | Elevated Triglycerides | Reduced  HDL-C | Elevated Blood Pressure | Elevated Fasting Glucose | METS  (Features Only) | METS  (Probability Only) | METS  (Combination) |
| LR | Internal | Brier score | 0.3449 | 0.2533 | 0.2357 | 0.2431 | 0.2548 | 0.2386 | 0.1835 | 0.2396 |
|  |  | ECE | 0.3424 | 0.1229 | 0.1316 | 0.2096 | 0.1869 | 0.1817 | 0.1413 | 0.1932 |
|  |  | MCE | 0.3424 | 0.1229 | 0.2438 | 0.3753 | 0.3560 | 0.3886 | 0.3344 | 0.4054 |
|  | External | Brier score | 0.2805 | 0.2528 | 0.2355 | 0.2479 | 0.2947 | 0.2443 | 0.1874 | 0.2463 |
|  |  | ECE | 0.2775 | 0.1178 | 0.1590 | 0.1659 | 0.4436 | 0.1791 | 0.1710 | 0.2167 |
|  |  | MCE | 0.2775 | 0.1178 | 0.3983 | 0.3072 | 0.4592 | 0.4078 | 0.3496 | 0.4232 |
| RF | Internal | Brier score | 0.3548 | 0.3531 | 0.2501 | 0.3186 | 0.3152 | 0.3287 | 0.1692 | 0.1672 |
|  |  | ECE | 0.0000 | 0.3346 | 0.1985 | 0.2763 | 0.2766 | 0.3103 | 0.0856 | 0.0877 |
|  |  | MCE | 0.0000 | 0.3346 | 0.2498 | 0.2763 | 0.2766 | 0.3103 | 0.1869 | 0.1488 |
|  | External | Brier score | 0.2895 | 0.3572 | 0.3616 | 0.3292 | 0.1038 | 0.2608 | 0.1681 | 0.1571 |
|  |  | ECE | 0.0000 | 0.3394 | 0.3367 | 0.2909 | 0.0006 | 0.2337 | 0.1150 | 0.0344 |
|  |  | MCE | 0.0000 | 0.3394 | 0.3367 | 0.2909 | 0.0006 | 0.2337 | 0.2063 | 0.0590 |
| XGB | Internal | Brier score | 0.0814 | 0.2084 | 0.2213 | 0.1769 | 0.2033 | 0.1577 | 0.1559 | 0.1557 |
|  |  | ECE | 0.0202 | 0.0742 | 0.1522 | 0.0666 | 0.0744 | 0.0368 | 0.0272 | 0.0223 |
|  |  | MCE | 0.0518 | 0.1312 | 0.1938 | 0.1128 | 0.1713 | 0.1237 | 0.0698 | 0.0680 |
|  | External | Brier score | 0.1160 | 0.2178 | 0.2194 | 0.2109 | 0.1630 | 0.1608 | 0.1611 | 0.1603 |
|  |  | ECE | 0.0797 | 0.0447 | 0.0526 | 0.0412 | 0.2995 | 0.0991 | 0.1131 | 0.1067 |
|  |  | MCE | 0.1708 | 0.1012 | 0.0761 | 0.0717 | 0.5634 | 0.1955 | 0.2702 | 0.2749 |
| MLP | Internal | Brier score | 0.2041 | 0.3265 | 0.2218 | 0.2476 | 0.3647 | 0.2722 | 0.1830 | 0.2111 |
|  |  | ECE | 0.1868 | 0.3384 | 0.1242 | 0.1122 | 0.3793 | 0.2151 | 0.1411 | 0.2074 |
|  |  | MCE | 0.3984 | 0.4315 | 0.2342 | 0.1653 | 0.8477 | 0.2389 | 0.2352 | 0.4046 |
|  | External | Brier score | 0.1854 | 0.3251 | 0.2531 | 0.2553 | 0.5494 | 0.2707 | 0.1897 | 0.2151 |
|  |  | ECE | 0.1894 | 0.3195 | 0.1905 | 0.0904 | 0.5966 | 0.2756 | 0.1754 | 0.1668 |
|  |  | MCE | 0.3636 | 0.4713 | 0.2795 | 0.1185 | 0.8226 | 0.3525 | 0.3687 | 0.2730 |
| TAB | Internal | Brier score | 0.0829 | 0.2063 | 0.2235 | 0.1793 | 0.2042 | 0.1593 | 0.1568 | 0.1577 |
|  |  | ECE | 0.0152 | 0.0681 | 0.1299 | 0.0713 | 0.0622 | 0.0545 | 0.0265 | 0.0331 |
|  |  | MCE | 0.0575 | 0.1466 | 0.1886 | 0.1358 | 0.1623 | 0.0955 | 0.0911 | 0.1234 |
|  | External | Brier score | 0.1129 | 0.2155 | 0.2161 | 0.2122 | 0.1633 | 0.1628 | 0.1637 | 0.1620 |
|  |  | ECE | 0.0625 | 0.0490 | 0.0270 | 0.0433 | 0.3244 | 0.1073 | 0.1081 | 0.1106 |
|  |  | MCE | 0.1428 | 0.1797 | 0.0480 | 0.1088 | 0.8252 | 0.1710 | 0.2477 | 0.2843 |
| **Abbreviations**: LR, logistic regression; RF, random forest; XGB, extreme gradient boosting; MLP, multi-layer perceptron; TAB, tabnet; METS, metabolic syndrome; HDL-C, high-density lipoprotein cholesterol; ECE, expected calibration error; MCE, maximum calibration error.  ***Notes***: The models in this table were calibrated using the final calibration method (sigmoid or isotonic) selected from 5-fold cross-validation results. Internal was conducted using The KNHANES 2022 data. External was conducted using the first follow-up of the Korean Genome and Epidemiology Study. Brier score, ECE, and MCE were measured for each validation of dataset, calibration method, and target. | | | | | | | | | | |
