# Supplementary material for: Development of a Predictive Model for Metabolic Syndrome Using Noninvasive Data and its Cardiovascular Disease Risk Assessments: Multicohort Validation Study
Source: J Med Internet Res. 2025 May 2;27:e67525. doi: 10.2196/67525 (PMC12084770; doi:10.2196/67525)
Supplement: Multimedia Appendix 5 [file jmir_v27i1e67525_app5.docx]

| Variable^a^ | | Dataset | | |
| --- | --- | --- | --- | --- |
|  |  | Internal | External 1 | External 2 |
| Male | Accuracy | 0.7267 | 0.7495 | 0.7367 |
|  | F1 Score | 0.7282 | 0.7607 | 0.7575 |
|  | AUROC | 0.8087 | 0.8137 | 0.8088 |
|  | AUPRC | 0.7259 | 0.6192 | 0.5084 |
| Female | Accuracy | 0.7911 | 0.7543 | 0.7509 |
|  | F1 Score | 0.7904 | 0.7564 | 0.7596 |
|  | AUROC | 0.8646 | 0.8143 | 0.8124 |
|  | AUPRC | 0.7118 | 0.6474 | 0.5928 |
| **Abbreviations**: AUROC, area under the receiver operating characteristic curve; AUPRC, area under the precision-recall curve.  ***Notes***: ^a^ This table presents the sex‐stratified performance of the best‐performing model for metabolic syndrome in the external validation datasets. | | | | |
