# Supplementary figures and images for: Development of a Predictive Model for Metabolic Syndrome Using Noninvasive Data and its Cardiovascular Disease Risk Assessments: Multicohort Validation Study
Source: J Med Internet Res. 2025 May 2;27:e67525. doi: 10.2196/67525 (PMC12084770; doi:10.2196/67525)

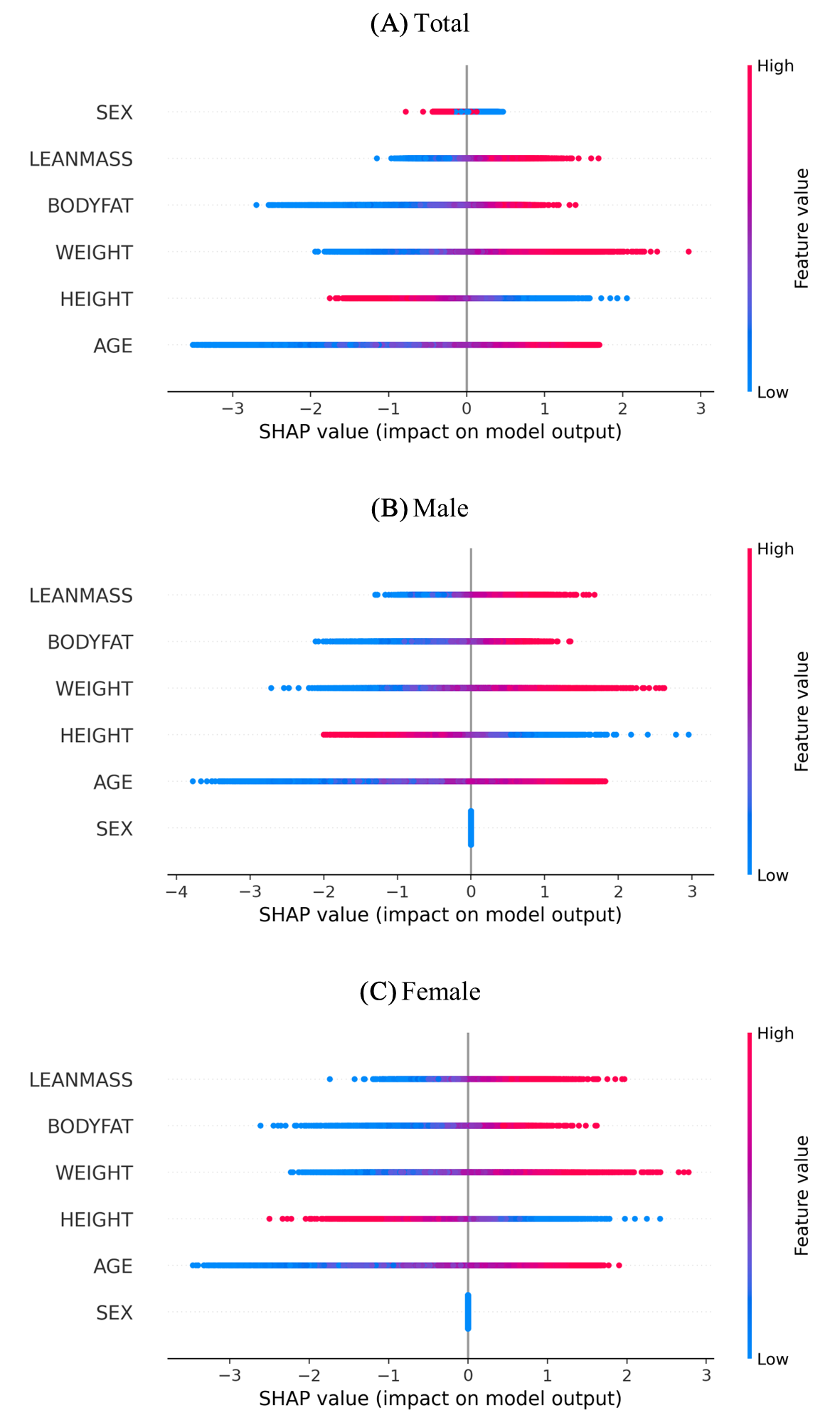

Supplement: Multimedia Appendix 6 [file jmir_v27i1e67525_app6.docx]

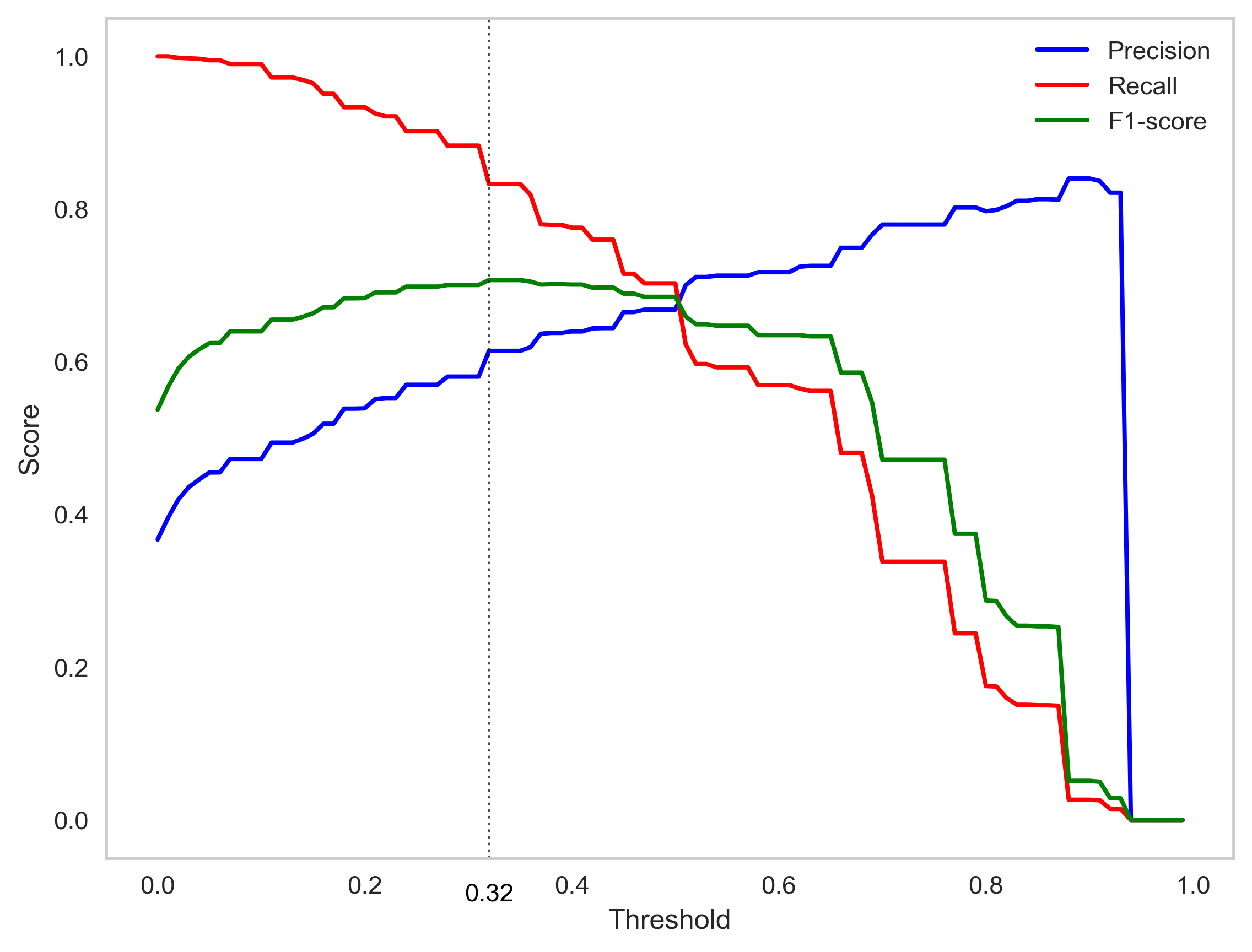

Supplement: Multimedia Appendix 7 [file jmir_v27i1e67525_app7.docx]

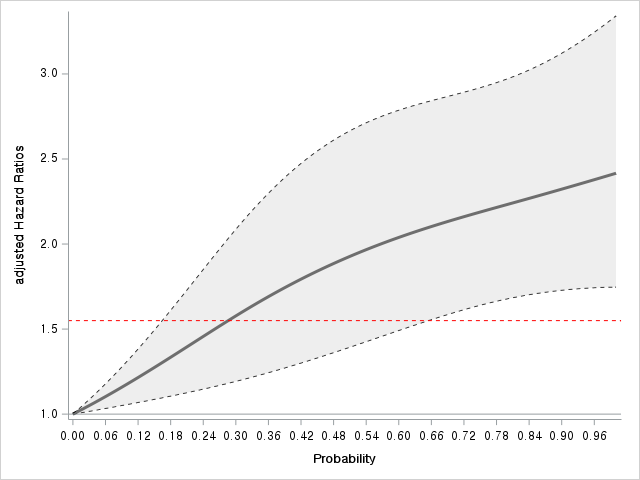

Supplement: Multimedia Appendix 12 [file jmir_v27i1e67525_app12.docx]

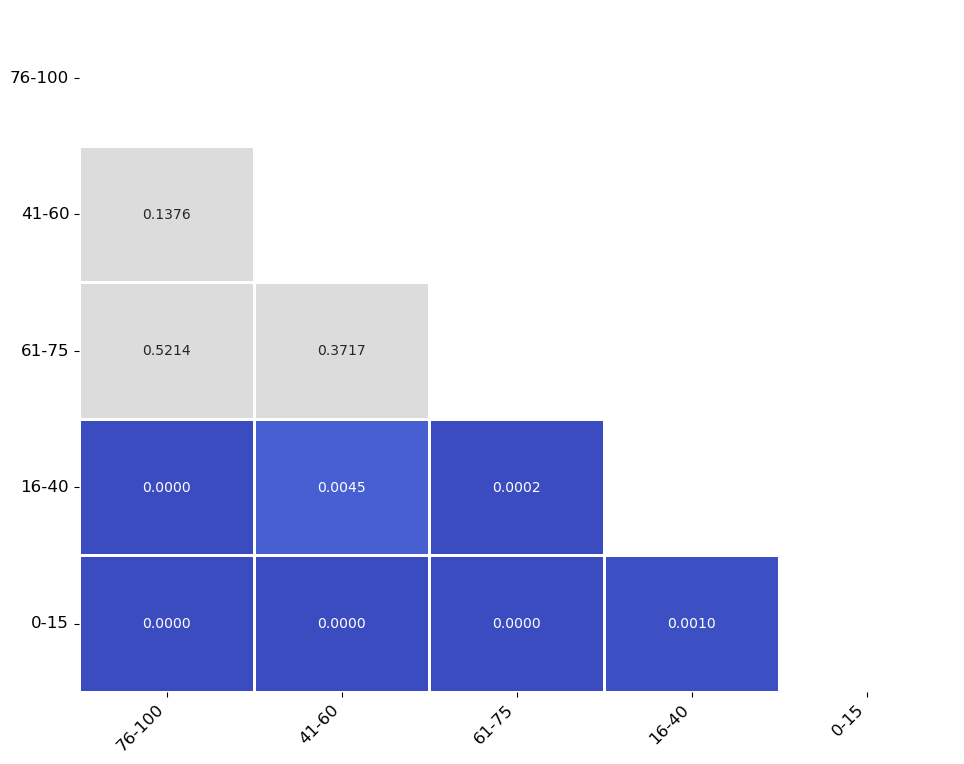

Supplement: Multimedia Appendix 13 [file jmir_v27i1e67525_app13.docx]
