# Supplementary material for: Development of a Predictive Model for Metabolic Syndrome Using Noninvasive Data and its Cardiovascular Disease Risk Assessments: Multicohort Validation Study
Source: J Med Internet Res. 2025 May 2;27:e67525. doi: 10.2196/67525 (PMC12084770; doi:10.2196/67525)
Supplement: Multimedia Appendix 8 [file jmir_v27i1e67525_app8.docx]

| **Cohort** | **Features** | **Total** | **Non-CVD** | **CVD** | ***P*-value** |
| --- | --- | --- | --- | --- | --- |
| Real METS  (External  validation 1) |  | ***n* = 2,035** | ***n* = 1,777** | ***n* = 258** |  |
|  | Incidence of METS at the second follow-up ^a^ | 1,029 (61.10) | 892 (60.15) | 137 (68.16) | .04^b^ |
|  | Age, year | 54.58±8.52 | 54.33±8.54 | 56.26±8.22 | <.001^b^ |
|  | Female, person | 1,188 (58.38) | 1,036 (58.30) | 152 (58.91) | .91 |
|  | BMI, kg/m^2^ | 26.62±2.88 | 26.60±2.93 | 26.76±2.58 | .41 |
|  | Body Fat, % | 30.23±6.49 | 30.16±6.55 | 30.77±6.04 | .16 |
|  | Alcohol, person | 859 (42.21) | 756 (42.54) | 103 (39.92) | .47 |
|  | Smoke, person | 433 (21.28) | 375 (21.10) | 58 (22.48) | .67 |
|  | PA, kcal/week | 11926±7622 | 11914±7623 | 12007±7629 | .85 |
|  | Income |  |  |  | .77 |
|  | - 200 | 1,427 (70.12) | 1,246 (70.12) | 181 (70.16) |  |
|  | 200 - 400 | 470 (23.10) | 408 (22.96) | 62 (24.03) |  |
|  | 400 - | 138 (6.78) | 123 (6.92) | 15 (5.81) |  |
| Real METS  (External  validation 2) |  | ***n* = 1,533** | ***n* = 1,368** | ***n* = 165** |  |
|  | History of METS at the first follow-up | 993 (64.77) | 870 (63.60) | 123 (74.55) | .007^b^ |
|  | Age, year | 56.04±8.60 | 55.82±8.59 | 57.85±8.52 | .004^b^ |
|  | Female, person | 913 (59.56) | 817 (59.72) | 96 (58.18) | .77 |
|  | BMI, kg/m^2^ | 26.76±2.86 | 26.77±2.90 | 26.73±2.48 | .88 |
|  | Change in BMI, kg/m^2^ | 0.22±1.06 | 0.22±1.07 | 0.22±0.99 | .92 |
|  | Body Fat, % | 30.17±6.54 | 30.16±6.58 | 30.33±6.18 | .75 |
|  | Change in Body Fat, % | 0.36±3.93 | 0.39±3.94 | 0.12±3.95 | .41 |
|  | Alcohol, person |  |  |  | .50 |
|  | Non-Alcoholic | 738 (48.14) | 652 (47.66) | 86 (52.12) |  |
|  | Abstinent | 88 (5.74) | 78 (5.70) | 10 (6.06) |  |
|  | Current Alcohol Use | 707 (46.12) | 638 (46.64) | 69 (41.82) |  |
|  | Smoke, person |  |  |  | .27 |
|  | Non-Smoker | 1,157 (75.47) | 1,037 (75.80) | 120 (72.73) |  |
|  | Former Smoker | 68 (4.44) | 63 (4.61) | 5 (3.03) |  |
|  | Current Smoker | 308 (20.09) | 268 (19.59) | 40 (24.24) |  |
|  | Income, million KRW |  |  |  | .49 |
|  | $<$ 2 | 1,053 (68.69) | 933 (68.20) | 120 (72.73) |  |
|  | $\geq$ 2, $<$ 4 | 359 (23.42) | 325 (23.76) | 34 (20.61) |  |
|  | $\geq$ 4 | 121 (7.89) | 110 (8.04) | 11 (6.67) |  |

**Abbreviations:** METS, metabolic syndrome; CVD, cardiovascular disease; BMI, body mass index; PA, physical activity.

***Notes:*** In Case ^a^, 351 individuals with missing Metabolic Syndrome data at the 2nd follow-up were excluded. ^b^ Indicates cases with *P* < 0.05.
