# Supplementary material for: Development of a Predictive Model for Metabolic Syndrome Using Noninvasive Data and its Cardiovascular Disease Risk Assessments: Multicohort Validation Study
Source: J Med Internet Res. 2025 May 2;27:e67525. doi: 10.2196/67525 (PMC12084770; doi:10.2196/67525)
Supplement: Multimedia Appendix 9 [file jmir_v27i1e67525_app9.docx]

| **Cohort** | **Features** | **HR (95% CI)** | ***P*-value** | **aHR (95% CI)** | ***P*-value** |
| --- | --- | --- | --- | --- | --- |
| External  Cohort 1 | Real METS |  | <.001^b^ |  | <.001^b^ |
|  | No | 1.00 (ref) |  | 1.00 (ref) |  |
|  | Yes | 1.91 (1.63–2.24) |  | 1.55 (1.29–1.86) |  |
|  | Predicted METS |  | <.001^b^ |  | <.001^b^ |
|  | No | 1.00 (ref) |  | 1.00 (ref) |  |
|  | Yes | 1.96 (1.66–2.31) |  | 1.57 (1.23–2.01) |  |
| External  Cohort 2 | Real METS |  | <.001^b^ |  | <.001^b^ |
|  | No | 1.00 (ref) |  | 1.00 (ref) |  |
|  | Yes | 1.69 (1.40–2.04) |  | 1.45 (1.19–1.76) |  |
|  | Predicted METS |  | <.001^b^ |  | <.001^b^ |
|  | No | 1.00 (ref) |  | 1.00 (ref) |  |
|  | Yes | 1.75 (1.45–2.10) |  | 1.53 (1.20–1.94) |  |
| Real METS  (External  validation 1) | BMI, kg/m^2^ |  |  |  |  |
|  | <23 | 1.00 (ref) | - | 1.00 (ref) | - |
|  | >=23, <25 | 2.12 (1.15–3.92) | .02^b^ | 2.31 (1.24–4.29) | .008^b^ |
|  | >=25 | 2.06 (1.18–3.62) | .01^b^ | 2.32 (1.27–4.23) | .006^b^ |
|  | Body Fat, % |  |  |  |  |
|  | <Q25 | 1.00 (ref) |  | 1.00 (ref) | - |
|  | >=Q25, <Q75 | 1.67 (0.98–2.85) | .06 | 1.57 (0.89–2.75) | .12 |
|  | >=Q75 | 1.64 (0.95–2.81) | .07 | 1.45 (0.73–2.85) | .29 |
|  | PA, 10^3^kcal/week |  |  |  |  |
|  | <Q25 | 1.00 (ref) | - | 1.00 (ref) | - |
|  | >=Q25, <Q75 | 1.00 (0.73–1.36) | .98 | 0.99 (0.72–1.35) | .94 |
|  | >=Q75 | 0.95 (0.68–1.33) | .78 | 0.85 (0.61–1.19) | .34 |
|  | Drink |  | .31 |  | .42 |
|  | No | 1.00 (ref) |  | 1.00 (ref) |  |
|  | Yes | 0.88 (0.68–1.13) |  | 0.88 (0.65–1.19) |  |
|  | Smoke |  | .40 |  | .10 |
|  | No | 1.00 (ref) |  | 1.00 (ref) |  |
|  | Yes | 1.13 (0.85–1.52) |  | 1.35 (0.95–1.91) |  |
| Real METS  (External  validation 2) | History of METS at the first follow-up |  | .006^b^ |  | .02^b^ |
|  | No | 1.00 (ref) |  | 1.00 (ref) |  |
|  | Yes | 1.64 (1.16–2.33) |  | 1.57 (1.09–2.26) |  |
|  | BMI, kg/m^2^ |  |  |  |  |
|  | <23 | 1.00 (ref) | - | 1.00 (ref) | - |
|  | >=23, <25 | 1.89 (0.83–4.29) | .13 | 1.89 (0.83–4.29) | .15 |
|  | >=25 | 1.75 (0.82–3.76) | .15 | 1.75 (0.82–3.76) | .18 |
|  | Change in BMI, kg/m^2^ |  |  |  |  |
|  | <Q25 | 1.00 (ref) | - | 1.00 (ref) | - |
|  | >=Q25, <Q75 | 1.07 (0.71–1.62) | .74 | 1.07 (0.71–1.62) | .88 |
|  | >=Q75 | 1.07 (0.70–1.65) | .75 | 1.07 (0.70–1.65) | .98 |
|  | Body Fat, % |  |  |  |  |
|  | <Q25 | 1.00 (ref) | - | 1.00 (ref) | - |
|  | >=Q25, <Q75 | 0.92 (0.51–1.65) | .77 | 0.92 (0.51–1.65) | .55 |
|  | >=Q75 | 0.94 (0.53–1.70) | .84 | 0.94 (0.53–1.70) | .29 |
|  | Change in Body Fat, % |  |  |  |  |
|  | <Q25 | 1.00 (ref) | - | 1.00 (ref) | - |
|  | >=Q25, <Q75 | 0.89 (0.60–1.31) | .55 | 0.89 (0.60–1.31) | .56 |
|  | >=Q75 | 0.79 (0.52–1.20) | .27 | 0.79 (0.52–1.20) | .19 |
|  | Drink |  |  |  |  |
|  | Non-Alcoholic | 1.00 (ref) | - | 1.00 (ref) | - |
|  | Abstinent | 0.90 (0.47–1.73) | .75 | 0.90 (0.47–1.73) | .90 |
|  | Current Alcohol Use | 0.82 (0.60–1.12) | .21 | 0.82 (0.60–1.12) | .15 |
|  | Smoke |  |  |  |  |
|  | Non-Smoker | 1.00 (ref) | - | 1.00 (ref) | - |
|  | Former Smoker | 0.74 (0.30–1.81) | .51 | 0.74 (0.30–1.81) | .70 |
|  | Current Smoker | 1.34 (0.93–1.91) | .11 | 1.34 (0.93–1.91) | .053 |

METS, metabolic syndrome; CVD, cardiovascular disease; BMI, body mass index; PA, physical activity. Q, quantile; ref, reference. ^b^ Indicates cases with *P* < 0.05.
