# Supplementary material for: Development of a Predictive Model for Metabolic Syndrome Using Noninvasive Data and its Cardiovascular Disease Risk Assessments: Multicohort Validation Study
Source: J Med Internet Res. 2025 May 2;27:e67525. doi: 10.2196/67525 (PMC12084770; doi:10.2196/67525)
Supplement: Multimedia Appendix 10 [file jmir_v27i1e67525_app10.docx]

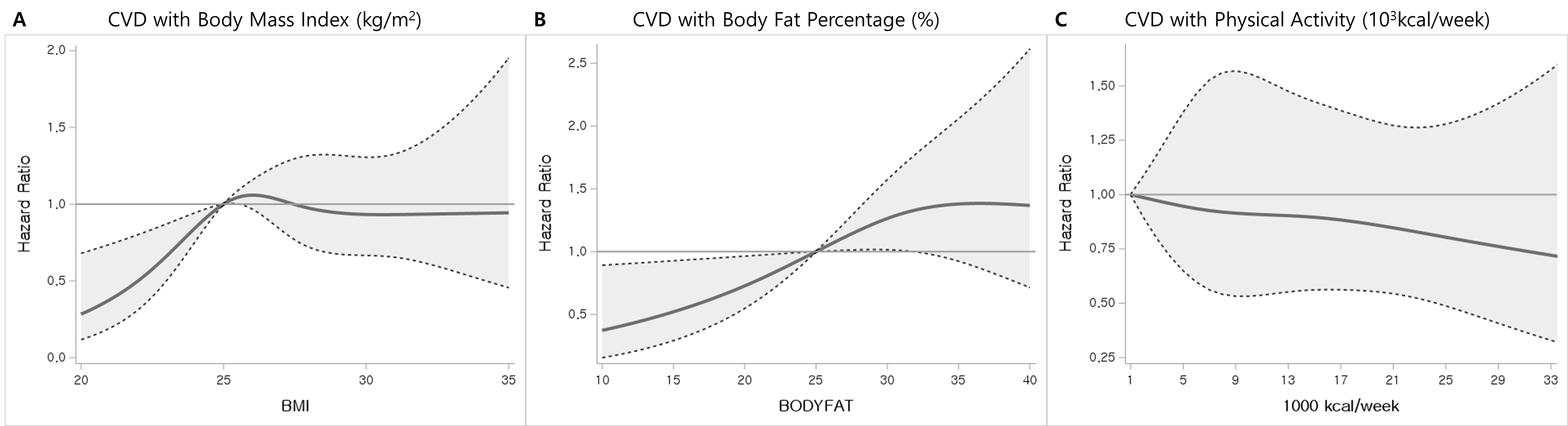


CVD indicates cardiovascular disease. Solid lines represent adjusted hazard ratios and shaded regions indicate 95% confidence intervals from restricted cubic spline regression. The cardiovascular disease hazard ratio was calculated for patients with metabolic syndrome. The hazard ratios were adjusted for sex, age, alcohol consumption, smoking status, and income level. A represents the hazard ratio based on body mass index, with a reference value of 25. B shows the hazard ratio based on body fat percentage, with a reference value of 25. C depicts the hazard ratio based on physical activity, with the reference value set at 1.
