# Supplementary material for: Development of a Predictive Model for Metabolic Syndrome Using Noninvasive Data and its Cardiovascular Disease Risk Assessments: Multicohort Validation Study
Source: J Med Internet Res. 2025 May 2;27:e67525. doi: 10.2196/67525 (PMC12084770; doi:10.2196/67525)
Supplement: Multimedia Appendix 11 [file jmir_v27i1e67525_app11.docx]

| **Risk group** | **Total Patients (%)** | **METS Patients (%)** | **CVD Patients (%)** |
| --- | --- | --- | --- |
| 0 - 15 | 2,023 | 102 (5.04) | 109 (5.39) |
| 16 - 40 | 2,226 | 416 (18.69) | 174 (7.82) |
| 41 - 60 | 1,134 | 407 (35.89) | 122 (10.76) |
| 61 - 75 | 1,017 | 567 (55.75) | 119 (11.70) |
| 76 - 100 | 750 | 543 (72.40) | 93 (12.40) |

**Abbreviations:** METS, metabolic syndrome; CVD, cardiovascular disease
